# An appraisal of laboratory models of androgenetic alopecia: A systematic review

**DOI:** 10.1002/ski2.15

**Published:** 2021-03-05

**Authors:** S. Ntshingila, N. P. Khumalo, M. Engel, A. T. Arowolo

**Affiliations:** ^1^ Hair and Skin Research Laboratory Division of Dermatology Department of Medicine Faculty of Health Sciences and Groote Schuur Hospital University of Cape Town Cape Town South Africa; ^2^ Department of Medicine Faculty of Health Sciences and Groote Schuur Hospital University of Cape Town Cape Town South Africa

## Abstract

**Background:**

Androgenetic alopecia (AGA) is the most common form of non‐scarring alopecia in humans. Several studies have used different laboratory models to study the pathogenesis and interventions for AGA. These study models have proved beneficial and have led to the approval of two drugs. However, the need to build on existing knowledge remains by examining the relevance of study models to the disease.

**Objective:**

We sought to appraise laboratory or pre‐clinical models of AGA.

**Method:**

We searched through databases (PubMed, ScienceDirect, Web of Science, World CAT, Scopus and Google Scholar) for articles on AGA‐related studies from 1942 to March 2019 with a focus on study models.

**Results:**

The search rendered 101 studies after screening and deduplication. Several studies (70) used in vitro models, mostly consisting of two‐dimensional monolayer cells for experiments involving the characterization of androgen and 5‐alpha reductase (5AR) and inhibition thereof, the effects of dihydrotestosterone (DHT) and biomarker(s) of AGA. Twenty‐seven studies used in vivo models of mice and monkeys to investigate DHT synthesis, the expression and inhibition of 5AR and hair growth. Only four studies used AGA‐related or healthy excisional/punch biopsy explants as ex vivo models to study the action of 5AR inhibitors and AGA‐associated genes. No study used three‐dimensional [3‐D] organoids or organotypic human skin culture models.

**Conclusion:**

We recommend clinically relevant laboratory models like human or patient‐derived 3‐D organoids or organotypic skin in AGA‐related studies. These models are closer to human scalp tissue and minimize the use of laboratory animals and could ultimately facilitate novel therapeutics.

## Graphical abstract

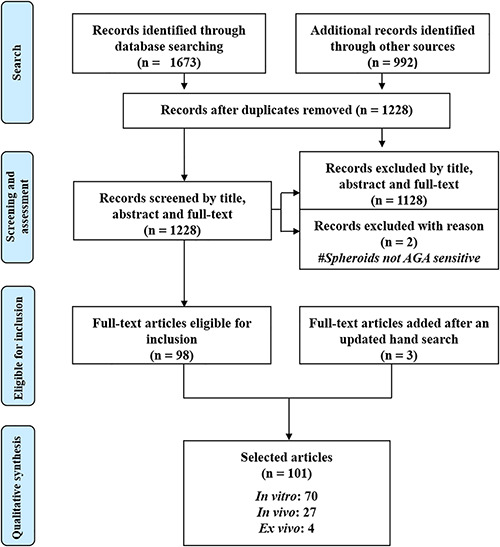

The current laboratory models of Androgenetic alopecia (AGA) are not representative of the disorder. Thus, this study evaluated all the laboratory models of AGA used in the last seven decades.This article recommends the use of patient‐derived 3‐D skin/ scalp cells or tissue culture models for future research studies on AGA instead of non‐representative 2‐D human and animal models.

1

**What's already known about this topic**

- Laboratory models are used to study the aetiology, pathogenesis and the efficacy of therapeutic interventions for AGA. These models, though beneficial, have only resulted in two FDA‐approved drugs for the treatment of AGA, which may be partly due to the lack of disease‐related models.

**What's new or what does this study add?**

- This systematic review summarizes published evidence of laboratory models used in AGA‐related research and revealed the scarcity of studies that use human or patient‐derived 3‐D tissue culture models. These models are more suitable and more physiologically representative than 2‐D cell culture and animal models for pre‐clinical research. Thus, this article could guide the selection of appropriate study models for future research, and thus facilitate research translation.

## 2 BACKGROUND

Androgenetic alopecia (AGA) is the most common form of non‐scarring hair loss in humans.^1^, ^2^, ^3^ The prevalence of AGA varies between races and ethnicities.^1^ This disparity is attributed to the different methods of measuring prevalence, making it difficult to compare studies.^1^ ^,^ ^2^ Nonetheless, about 50% of men of European descent are affected by the age of 50 years; this proportion increases to 90% with age.^1^ ^,^ ^4^ ^,^ ^5^ Furthermore, AGA is estimated to affect about 19% of women of European ancestry, while the prevalence and severity of AGA are considered low in Asian and African men.^1^ ^,^ ^4^ ^,^ ^6^

In the early 1940s, Hamilton proved that genetic predisposition and male hormone stimulation are prerequisites in AGA development.^7^ After this discovery, several AGA models were created to delineate the pathophysiology and evaluate the effectiveness of novel therapeutics using both laboratory (in vitro, in vivo and ex vivo) and non‐laboratory models. These models, though beneficial, however, have limitations since most are not fully representative of AGA. Also, available information on the molecular pathology and mechanism of AGA action is modest and thus hampers the progression of potential new treatments to human clinical trials. This problem may explain why only two drugs (Minoxidil, a vasodilator, and Finasteride, an alpha two reductase inhibitor) are currently approved and available for AGA treatment. Therefore, it is imperative to build upon existing models of AGA to facilitate the discovery of novel therapeutics.

This systematic review aims to appraise all AGA‐related study models published to date for the testing of AGA interventions. However, not intended to evaluate the efficacy of AGA treatments.

## 3 METHODS

The protocol for this study was registered and published in the International Prospective Register of Systematic Reviews (reg. number CRD42018107182; http://www.crd.york.ac.uk/PROSPERO).

### 3.1 Search strategy

The Medical Search Headings terms used for the search were Androgenetic alopecia, Androgenic Alopecia, Hair Loss, and Male pattern baldness. Other search terms included In vitro, In vivo, Ex vivo, Finasteride, Minoxidil, Proliferation, Hair loss culture models and Xenografts, Tissue culture, Plant extracts, Hair shedding, Hair scalp biopsies, Induced alopecia, Cell differentiation, Cell lines, Primary cells, 2‐D Models, 3‐D Models, and Significant hair growth. The ‘NOT’ Boolean operator was used to exclude reviews, ‘transplant studies’, ‘human studies’, and ‘clinical studies’ (Table S1).

### 3.2 Information sources

There was a search for articles on publication databases (PubMed, ScienceDirect, Web of Science, World CAT, Scopus and Google Scholar) from January 1942 until March 2019. A search strategy was developed for PubMed and adapted as per the requirements of other databases. A hand search in Google Scholar and Science Direct supplemented the search. The review also looked at unpublished articles from conferences and libraries (dissertations).

### 3.3 Inclusion criteria

The inclusion criteria for the articles selected are as follows: preclinical laboratory studies and models (in vitro, in vivo and ex vivo) published in the English language. Our search excluded all articles involving human, clinical, diagnostic, drugs/phytochemicals efficacy and hair or cell transplant studies. Search results were filtered to include the research studies relevant to this review as per the pre‐decided inclusion criteria.

### 3.4 Data extraction and synthesis

We used a predesigned template adapted from Cochrane to populate the data extracted from the selected studies (Table S2). We analysed these data based on certain variables: the type of model (i.e., in vitro, ex vivo and in vivo), cell‐types and techniques. We grouped research articles with similar variables.

### 3.5 Quality assessment and risk of bias

The selected articles' quality was assessed by two authors (SN and AA) using a predesigned template validated by three independent assessors (Table S3). The quality assessors scored 10 similar questions, compared scoring results and any disagreements resolved through consensus. The articles were assessed against three questions, as follows:

1. Is the model used in the study appropriate or relevant (i.e., the use of patients' or control direct tissues or biopsies, patient‐derived primary or immortalized two dimensional [2‐D] or three dimensional [3‐D] cells/cell lines)? Studies that used patient‐related or human tissue instead of animal tissue as models had high scores.
2. Do the techniques and methods of analysis used in the study adequately address the research question?
3. Are the results from the research study validated by more than one research technique and are the results reproducible (i.e., the number of independent experiments)? Articles with results confirmed by three or more techniques, and a minimum of three independent experiments, scored higher than studies that used two or one methods.

The maximum score per question was five points.

## 4 RESULTS

### 4.1 Study selection

**FIGURE 1:**
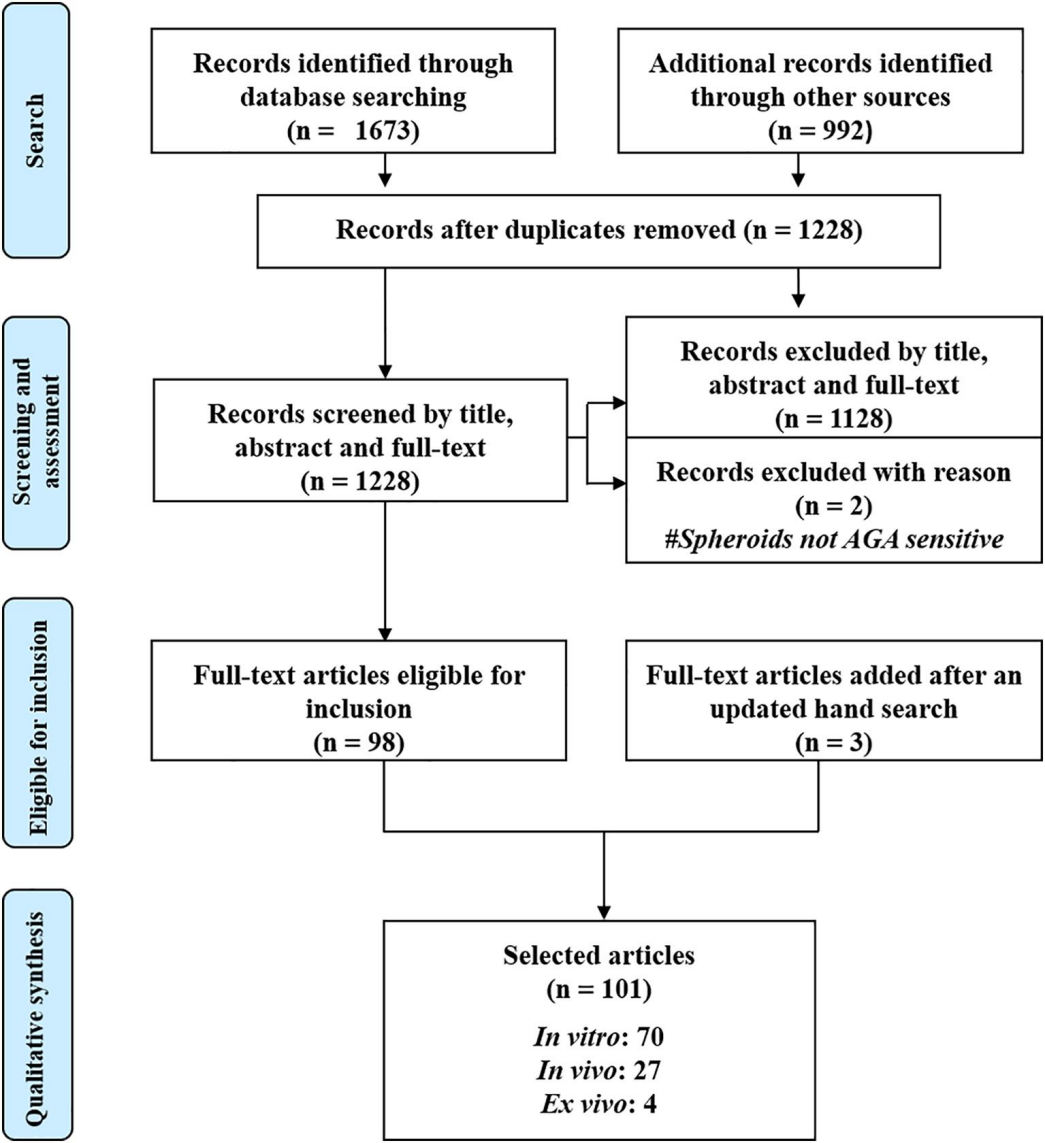
A PRISMA diagram illustrating the search, screening and assessment strategy of articles reviewed, and qualitative synthesis of the selected articles

Our initial search rendered 1228 related articles after removing duplicates. A further review of the full‐text articles based on set eligibility criteria excluded 1128 and an additional two with the reason (spheroids but unrelated to AGA). Three more research papers retrieved from an updated hand search supplement the remaining 98 articles, thus bringing the entire selected research articles to 101. These articles included studies involving 70 in vitro, 4 ex vivo and 27 in vivo models (Figure 1).

### 4.2 Data extraction and synthesis of included studies

**FIGURE 2:**
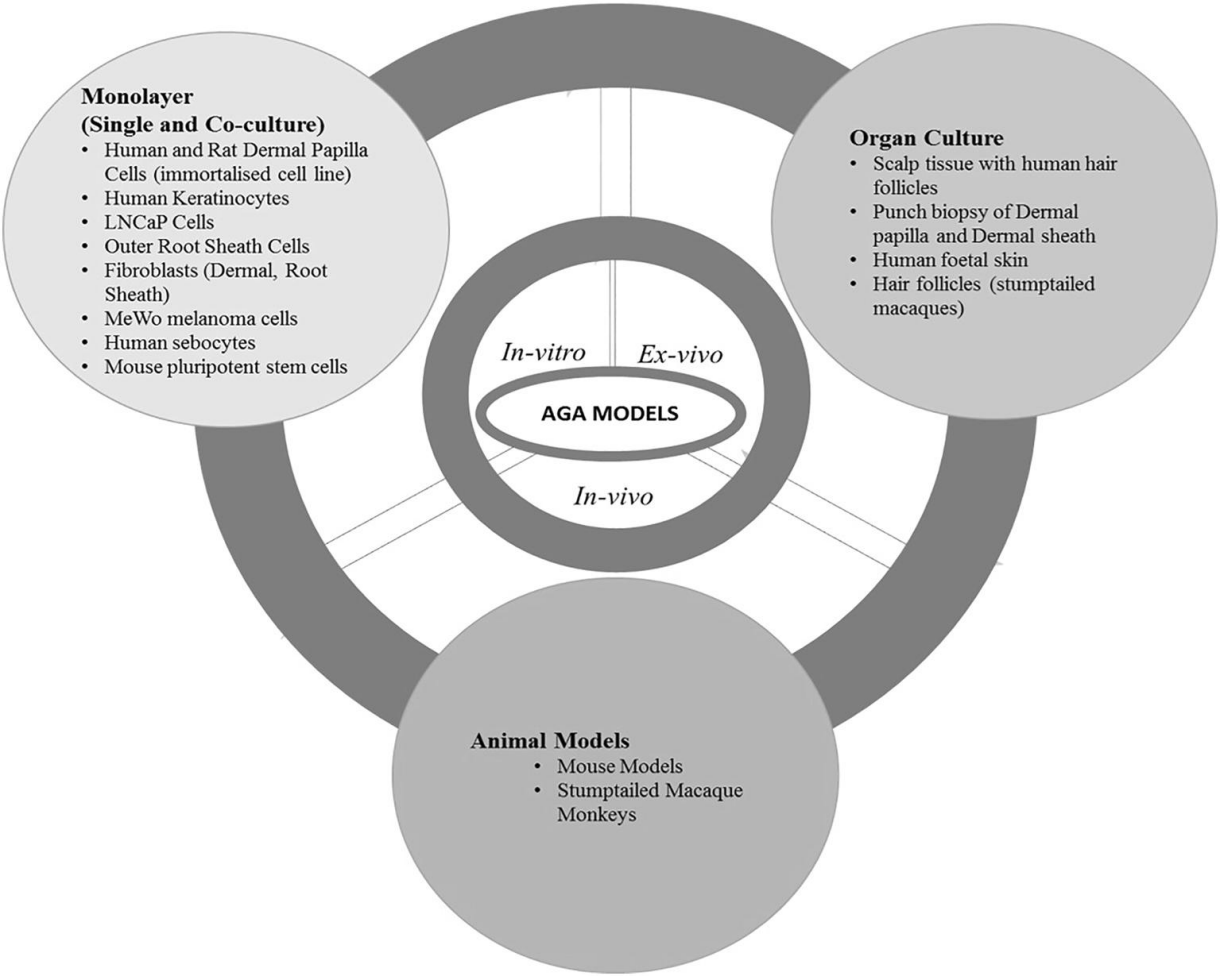
An overview of the in vitro, ex vivo and in vivo model of androgenetic alopecia (AGA) included in the review

A summary of the study models from the included studies (i.e., in vitro, ex vivo and in vivo) is illustrated (Figure 2) and grouped under the following headings (Table 1): Study objective(s), Models, Techniques and Outcomes. From the 101 articles, 15 demonstrated the requirement of androgens for AGA development using in vitro and ex vivo models with different cell‐based experimental techniques. These techniques include cell proliferation assays involving androgen‐induced overgrowth and androgen receptor antagonism.^3^, ^4^, ^5^, ^6^, ^7^, ^8^, ^9^, ^10^, ^11^, ^12^, ^13^, ^14^, ^15^, ^16^, ^17^ Twenty‐four studies investigated gene expression profiling^18^, ^19^, ^20^, ^21^, ^22^, ^23^, ^24^, ^25^, ^26^, ^27^, ^28^ and markers for AGA.^29^, ^30^, ^31^, ^32^, ^33^, ^34^, ^35^, ^36^, ^37^, ^38^, ^39^, ^40^, ^41^ Fourteen studies focused on the action of 5‐alpha reductase (5AR), which converts the androgen (testosterone) into dihydrotestosterone (DHT) and the inhibition of this enzyme^42^, ^43^, ^44^, ^45^, ^46^, ^47^, ^48^, ^49^, ^50^, ^51^, ^52^, ^53^, ^54^, ^55^ using enzymatic, radiochemical and cell proliferation assays. Thirteen studies used the same techniques to investigate the role of other enzymes in the mediation of AGA.^4^ ^,^ ^6^ ^,^ ^10^ ^,^ ^43^ ^,^ ^56^, ^57^, ^58^, ^59^, ^60^, ^61^, ^62^, ^63^, ^64^, ^65^ To find alternative treatments for AGA, four studies investigated the effect of plant extracts and phytocompounds on the inhibition of 5AR type 2 using the dermal papilla cells (DPCs) proliferation assay.^43^ ^,^ ^45^ ^,^ ^48^ ^,^ ^53^ Only three studies investigated efficient drug delivery methods, such as nanotechnology.^66^, ^67^, ^68^ In contrast, 27 articles used mice/mice xenografts, rats and monkeys as in vivo models to study the efficacy of Finasteride, Minoxidil and plant extracts/compounds in the treatment of AGA. Different assays were used, including 5AR enzymatic action on androgens, DHT effect on hair and hair appendages growth experiments.^10^ ^,^ ^21^ ^,^ ^46^ ^,^ ^50^, ^51^, ^52^ ^,^ ^64^ ^,^ ^69^, ^70^, ^71^, ^72^, ^73^, ^74^, ^75^, ^76^, ^77^, ^78^, ^79^, ^80^, ^81^, ^82^, ^83^, ^84^, ^85^, ^86^, ^87^, ^88^, ^89^, ^90^, ^91^, ^92^

**TABLE 1:** A summary of the extracted data from the selected and reviewed research articles

	Study objective(s)	Models	Techniques	Outcomes	References
					No. studies
In vitro	Androgens are required for AGA mediation	DPC PC3 cells HaCaTs LNCaP	Cell proliferation assays; Androgen‐induced overgrowth assay; Androgen receptor antagonism assay	The models proved that androgens are required for the mediation of AGA	^3^, ^4^, ^5^, ^6^, ^7^, ^8^, ^9^, ^10^, ^11^, ^12^, ^13^, ^14^, ^15^, ^16^, ^17^ 15 studies
	The role of phytocompounds	DPC	5AR inhibition assays; DPC proliferation assays;	The phytochemical compounds tested inhibited the action of 5AR‐ type 2 and regulated multiple genes associated with AGA (IGF‐1, DKK‐1, TGF‐β1, IL‐1α, VEGF)	^43^, ^45^, ^48^, ^53^ Four studies
In vitro and Ex vivo	The action of 5 α‐reductase and inhibitors	HEK293 DPC LNCaP cells. Excisional biopsies (whole skin organ culture of DPC) Hair follicles (DPC) Mouse mammary carcinoma Shionogi S115 cell line Human serum from blood transfusion SZ95 sebocytes HaCaTs MeWo melanoma cells	5AR enzymatic assay A radiochemical assay Cell proliferation assay Androgen receptor antagonism assay Standard Bradford protein assay	5AR concentrated in DPC changes testosterone into DHT in the cytoplasm	^42^, ^43^, ^44^, ^45^, ^46^, ^47^, ^48^, ^49^, ^50^, ^51^, ^52^, ^53^, ^54^, ^55^ 14 studies
				Enzymes responsible for the intracellular activation and inactivation of androgens showed mRNA expression patterns that correlated with the enzyme activities with and without use of selective enzyme inhibitors respectively.	^4^, ^6^, ^10^, ^43^, ^56^, ^57^, ^58^, ^59^, ^60^, ^61^, ^62^, ^63^, ^64^, ^65^ 14 studies (2‐ ex vivo)
	The role of genes: Expression studies and profiling Markers for AGA	Excisional biopsies (whole skin organ culture of AGA affected skin) HaCaTs Immortalized DPC lines 3D AGA Spheroid HFSC Punch biopsies	AGA gene mapping and gene expression graphs (GEG) across genome microRNA expression profile from balding and non‐balding scalp DNA microarray Cell proliferation, Immunohistochemical staining (2D RP‐RP) LC‐MS/MS	These studies discovered genes that are responsible for AGA and genes that promote hair growth. These studies identified the pathways for AGA; genes and proteins regulated in the presence of AGA.	^18^, ^19^, ^20^, ^21^, ^22^, ^23^, ^24^, ^25^, ^26^, ^27^, ^28^ 11 studies (1‐ ex vivo) ^29^, ^30^, ^31^, ^32^, ^33^, ^34^, ^35^, ^36^, ^37^, ^38^, ^39^, ^40^, ^41^ 13 studies
	Efficient delivery methods of drugs	Flutamide‐loaded SLNs Excised rat skin	Hot melt homogenization method. Drug permeation and accumulation *S.cerevisiae* model	The resultant PLGA nanoparticles were an efficient encapsulation system for FNS. No toxic effects observed from the *S. cerevisiae* model	^66^, ^67^, ^68^ Three studies (1‐ ex vivo)
In vivo	The use of finasteride, minoxidil, plant extracts and plant compounds for treatment of AGA	C57BL/6 Specific pathogenic free Kunming mice Male Sprague Dawley rats Male Wister/ST rats C57BL/6NCrSlc strain mice C3H/He strain mice B6CBACF1/J female mice Male Swiss albino mice Stumptailed macaque PHFCs HaCaTs	HaCaTs treated with LSESr Hair loss mouse model MTS assays (cell viability assay)	Increased density, weight and thickness of mouse hair. Also, a remarkable increase in size and shape of hair follicles and anagen/telogen ratio Increased cell viability and proliferation in cell‐based studies Inhibition of 5AR activity—inhibiting the DHT synthesis mRNA and protein expression of anagen maintaining growth factors	^10^, ^21^, ^46^, ^50^, ^51^, ^52^, ^64^, ^69^, ^70^, ^71^, ^72^, ^73^, ^74^, ^75^, ^76^, ^77^, ^78^, ^79^, ^80^, ^81^, ^82^, ^83^, ^84^, ^85^, ^86^, ^87^, ^88^, ^89^, ^90^ 27 studies

Abbreviations: AGA, androgenetic alopecia; DKK‐1, Dickkopf WNT signalling pathway inhibitor 1; DPC, dermal papilla cells; FNS, finasteride; GEG, gene expression graphs; HaCaTs cells, human keratinocytes; HFSC, hair follicle stem cells; IGF‐1, insulin‐like growth factor‐1; IL‐1α, interleukin‐1; LNCaP cells, prostate adenocarcinoma cells; LSESr, lipidosterolic extract of *Serenoa repens*; MeWo, melanoma cells: human melanoma cell line; MTS, (3‐(4,5‐dimethylthiazol‐2‐yl)‐5‐(3‐carboxymethoxyphenyl)‐2‐(4‐sulfophenyl)‐2H‐tetrazolium); PC3 cells, human prostate cancer cell line; PHFCs, primary hair follicle fibroblast cells; SLN, solid lipid nanoparticles; SZ95 sebocytes, immortalized human sebaceous gland cell lines; TGF‐β1, transforming growth factor beta1; VEGF, vascular endothelial growth factor.

### 4.3 Quality assessment of included studies

**FIGURE 3:**
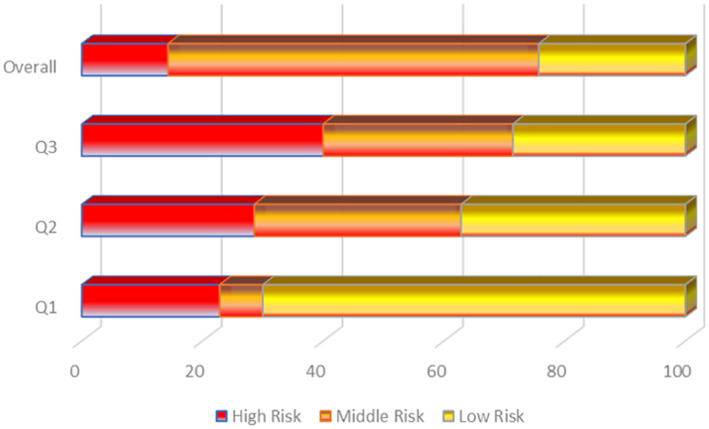
A quality assessment chart indicating the risk bias contained in the studies of the selected articles. Q1–Q3 = Assessment questions. Q1 = Is the model used in the study appropriate or relevant (i.e., the use of patients' or control direct tissues or biopsies, patient‐derived primary or immortalized 2‐D or 3‐D cells/cell lines)? Q2 = Do the techniques and methods of analysis used in the study adequately address the research question? Q3 = Are the results from the research study validated by more than one research technique, and are the results reproducible (i.e., the number of independent experiments)?

The overall results show a relatively high percentage (62%) of articles with a middle‐risk score (Figure 3). The evaluation of these articles revealed that 75% used appropriate models, while 25% of the studies having a high‐risk score on the reliability of the models. The models used are monolayer (single or co‐culture) of immortalized cell lines, human skin tissue, or mice/mice xenografts, or stump‐tailed macaques. The articles had clear objectives or research questions, with methods and procedures that precisely addressed these objectives. About 38% of the selected studies had not validated experimental results with more than one technique.

## 5 DISCUSSION

This systematic review, which sought to appraise models used in preclinical AGA research, rendered three groups of models: in vitro (*n* = 70), ex vivo (*n* = 4) and in vivo (*n* = 27).

### 5.1 In vitro 2‐D models

The use of in vitro models is fundamental to all biomedical studies. The advancements made in studying cells, bacteria and viruses are often the first strides into understanding in vivo conditions. The in vitro models from included studies were 2‐D monolayer (single or co‐culture cells) of immortalized or primary epithelial and mesenchymal cell lines. Immortalized cell lines are often used in research in the place of primary cells because they are cost‐effective, easy to use and provide an unlimited supply of material. These cells can also grow indefinitely in culture and bypass ethical concerns associated with animal and human tissue use. However, immortalized cell lines undergo significant mutations to become immortal, which can alter cellular physiology. Also, genetic changes can occur over multiple passages, leading to phenotypic differences among isolates and unreproducible experimental data.

Examples of immortalized cells used in the reviewed articles were human prostate cancer (PC3) cells, human keratinocytes (HaCaTs), prostate adenocarcinoma (LNCaP) cells, human sebaceous gland (SZ95 sebocytes) cells, human melanoma cells, human embryonic kidney 293 cells and the Shionogi carcinoma‐115 cells.^4^ ^,^ ^6^ ^,^ ^10^ ^,^ ^43^ ^,^ ^56^, ^57^, ^58^, ^59^, ^60^, ^61^, ^62^, ^63^, ^64^, ^65^

Although the use of these epithelial cells provided useful data to study potential AGA therapeutics, they are not related to this disorder. Therefore, some studies used DPCs.^43^ ^,^ ^45^ ^,^ ^48^ ^,^ ^53^ Scalp DPCs are specialized mesenchymal cells of tissues affected by AGA that exist in the dermal papilla located at the basal layer of hair follicles. These cells play a pivotal role in the hair cycle. These cells were used as 2‐D in vitro models in androgen‐based and 5AR kinetics and inhibition assays to study the paradox of DHT, gene expression and AGA markers in two culture systems. The first system involved introducing growth factors (from keratinocytes‐conditioned growth medium with or without medium containing fibroblast growth factor) to cultured DPCs. The second system included the co‐culture of DPCs with effector cells (e.g., HaCaTs). Although these 2‐D models are instrumental in enhancing the understanding of AGA's molecular pathology and mechanism, there have been limitations associated with these 2‐D models. For instance, 2‐D models do not accurately recapitulate the tissue architecture and cellular mechanisms present in a whole skin, impeding advancement towards effective AGA therapeutics.

### 5.2 In vivo models

Twenty‐seven studies used either mice or rats and monkeys as in vivo models, the most common being C57BL/6, Kunming mice and male Sprague Dawley rats, male Wister/ST rats, C57BL/6NCrSlc strain mice, C3H/He strain mice, B6CBACF1/J female mice, male Swiss albino and human scalp hair grafted into nude mice (i.e., human scalp hair xenografts).^21^ ^,^ ^46^ ^,^ ^50^, ^51^, ^52^ ^,^ ^64^ ^,^ ^69^, ^70^, ^71^, ^72^, ^73^, ^74^, ^75^, ^76^, ^77^, ^78^, ^79^, ^80^, ^81^, ^82^, ^83^, ^84^, ^85^, ^86^, ^87^, ^88^, ^89^ ^,^ ^91^ ^,^ ^92^ These rodents are used in general as models in medical research due to genetic, biological and behavioural similarities to humans. More so, they are useful in replicating many human disorders.

The stump‐tailed macaque monkey was the only primate‐related in vivo model used in the reviewed studies.^10^ ^,^ ^88^ ^,^ ^90^ Stump‐tailed macaque was the preferred choice because they possess hereditary balding characteristics similar, in many respects, to those of AGA in humans. However, macaques are found only in Asia, and the cost, risk and near extinction compromise the use of this model. Hence, the number of studies using these monkeys has diminished over the years. Animal models are considered suitable for studying human diseases as their biological milieu resembles human homoeostatic conditions. However, the use of animals in research is subject to strict ethical guidelines and often laden by the high human translational failures, perhaps due to inadequate mimicry of human pathophysiology. Thus, 3‐D ex vivo models are fast becoming more prominent in understanding AGA.

### 5.3 Ex vivo models

Only four of the included studies were ex vivo, using either diseased (AGA) or normal excisional and punch biopsy explants (whole skin organ cultures).^6^ ^,^ ^23^ ^,^ ^60^ ^,^ ^67^ These models were used to study the action of 5AR inhibitors and the role of genes in AGA. Skin explants can maintain the cutaneous structure of the skin and, hence, allowing for studies, for example, that evaluate the effects of 5AR inhibitors on tissue morphology and gene expression. Although ex vivo organ culture is an easy‐to‐use and relatively cheap model, its use may be hampered by human ethical consideration and the availability of skin organ donors leading to insufficient skin specimens.

### 5.4 Risk of bias

A validity assessment evaluated the risk of bias in the models and experimental techniques used in the included studies. The overall results show a relatively high percentage of articles with a middle‐risk score. This observation may result from the low scores in the reliability of methods used and validation of results (we recommend a minimum of three techniques for validation). Result validation is a critical component in method evaluation because it reveals the specificity, accuracy, precision and, ultimately, the data's reliability. Validation is also essential to demonstrate that the model and technique used is suitable for the intended use. Many of the included studies (76%) were not exhaustive, with only 24% using three or more techniques to validate their results. Therefore, there is a need for continuity in work already done and the development of models with more relevant technologies.

Assessing the risk of bias of studies contained in the body of evidence is foundational and central to all systematic reviews to improve the transparency, consistency and scientific rigour of the research. Bias refers to factors that can confound the overall observations and conclusions of a study, which may lead to inappropriate recommendations. These discrepancies can result in wasted resources and loss of opportunities to discover effective therapies.

## 6 LIMITATIONS

None of the included studies used 3‐D models of AGA. The use of 3‐D AGA models (e.g., organ culture from either punch biopsies, skin tissues from patients [healthy or diseased] or cells isolated from tissue samples) has an advantage over 2‐D models. These models allow experimentation with human diseased tissues under controlled conditions than would be challenging to achieve in 2‐D models, which may prove unachievable with in vivo models, thus allowing for a more detailed cellular and molecular characterization. Interestingly, 2‐D models can be used to create 3‐D models. The 3‐D models can either be organoid cultures or be organotypic skin cultures. Organoids are in vitro‐derived 3‐D cell aggregates derived from primary tissue, which possess similar composition and architecture to primary tissue, and exhibit organ functionality. On the other hand, organotypic skin cultures use primary human cells, and cell culture inserts to recapitulate the stratified epidermal architecture of the skin to replicate the normal anatomy and physiology. This approach, especially with whole skin punch biopsies, allows for the in vitro maintenance of skin appendages, for example, hair follicles. Therefore, organotypic skin cultures may be instrumental in preclinical AGA research to validate the mechanisms of diseases and test the novel therapeutics.

## 7 CONCLUSION

There is substantial evidence on the use of various models in the study of many diseases, including AGA and the discovery of effective targeted therapies. AGA models to date have helped in the understanding of this disorder. Howbeit, most of these models are not ideal representatives of AGA in humans. Only four studies used whole human skin biopsies from patients (diseased and healthy), a closer approximation of AGA in humans. However, none of the studies used 3‐D organoids or organotypic skin culture models. Therefore, there is a need to improve these models with a focus on the use of diseased and healthy biopsies or cell lines isolated from these biopsies. These 3‐D models allow experimentation closer to humans, which may prove difficult or impossible with in vivo models while minimizing harm to animals. It is also vital to validate experiments with a minimum of three techniques. Finally, 3‐D experimental data should ultimately be tested in human clinical trials to achieve formidable progress towards fast and effective treatment solutions for AGA. A good translation of AGA preclinical research into human trials will ensure the discovery of new and more effective drugs.

## CONFLICT OF INTEREST

The authors have no conflict of interest to declare.

## Supporting information

Supplementary Material
