# Supplementary material for: An appraisal of laboratory models of androgenetic alopecia: A systematic review
Source: Skin Health Dis. 2021 Mar 5;1(2):e15. doi: 10.1002/ski2.15 (PMC9060143; doi:10.1002/ski2.15)
Supplement: Supplementary file 1 — Supplementary Material [file SKI2-1-e15-s001.docx]

**SUPPLEMENTARY TABLES**

**Table 1: Search Strategy**

| SEARCH TERMS |  |  |  |
| --- | --- | --- | --- |
| MeSH terms | #1 Androgenetic alopecia, Androgenic alopecia, Hair Loss, Male pattern baldness | | |
| All Fields: | #2 Invitro | #3 Finasteride | #4 Minoxidil |
|  | #5 NOT clinical studies | #6 Plant extracts | #7 tissue culture |
|  | #8 in vivo | #9 NOT transplants | #10 Induced alopecia |
|  | #11 Cell lines | #12 Significant hair growth | #13 NOT Review |
|  | #14 Cell differentiation | #15 Proliferation | #16 Hair loss culture models |
|  | #17 Control | #18 Ex vivo | #19 NOT Human Studies |
|  | #20 Hair shedding | #21 Biopsies |  |
| PICO SEARCH TERMS | The searches will be combined as follows: | | |
|  | *In vivo* | *In vitro* | *Ex vivo* |
| Population: |  | | |
|  | #1 and #3 and #5 and  #8 and #19 | #1 and #2 and #3 | #1 and #18 |
|  | #1 and #4 and #5 and #8, and #19 | #1 and #2 and #4 |  |
|  | #1 and #5 and #6 and #8 and #19 | #1 and #2 and #6 |  |
|  |  | #1 and #2 and #7 |  |
| Intervention: | | | |
|  | #1 and #5 and #8 and  #10 and #19 | #1 and #2 and #16, #1 and #2 and #11, #1 and #2 and #7 | #1 and #18 #1 and #18 and #21 #1 and #7 and #18 |
| Comparator | | | |
|  | #1 and #3 and #5 and #17  #1 and #4 and #5 and #17 | No strategy | No strategy |
| Outcome | | | |
|  | #1 and #5 and #8 and #12  #1 and #5 and #8 and #20 | #1 and #12 and #15 and #18 and #19 | #1 and #2 and #12 and #13 |
|  |  | #1 and #14 and #18  and #19 | #1 and #2 and #12  and #14 |
|  |  | #1 and #15 and #18  and #19 | #1 and #2 and #12  and #15 |

**Note: For the PICO terms each box is one combined search.**

**Table 2: Data extraction form**

| MODEL: | |
| --- | --- |
| Author |  |
| Journal |  |
| Publication year |  |
| Title |  |
| STUDY DESIGN |  |
| Model |  |
| Intervention |  |
| Outcomes Measure |  |
| CHARACTERISTICS OF THE MODEL |  |
| Biopsy site/ Building of organoid |  |
| Intervention |  |
| Condition Study |  |
| Outcome measure |  |
| Conclusion |  |
| CHARACTERISTICS OF INTERVENTION |  |
| PRIMARY OUTCOMES |  |

**Table 3: Quality assessment template**

Each section to be scored out of 5 points

|  |  | Score |  | Score |
| --- | --- | --- | --- | --- |
| How serious is the risk of bias? | |  | (additional score for human or animal model) | |
| Is the model appropriate for the study question? | 3-D Model | 3 | Human | 2 |
|  |  |  | Animal | 1 |
|  | 2-D Model | 2 | Human | 2 |
|  |  |  | Animal | 1 |
|  | Immortalized cell- lines | 1 | Human | 2 |
|  |  |  | Animal | 1 |
| Method of Analysis- does it address the objectives of the study? | | |  |  |
| Method of analysis used | Clear objective and/or research question | 2 |  |  |
|  | Number of relevant methods used |  | 3+ methods | 2 |
|  |  |  | 1-2 methods | 1 |
|  | Clarity of methods and procedures | 1 |  |  |
| Validation of results | Validated results using different and relevant techniques/ methods | 2 | 3+ methods | 2 |
|  |  |  | 1-2 methods | 1 |
|  | Legible presentation of results | 1 |  |  |
